# Case Report: A rare instance of acute appendicitis induced by Balantidium Coli parasitic infection

**DOI:** 10.3389/fped.2024.1410850

**Published:** 2024-05-13

**Authors:** Sergio Alzate-Ricaurte, Isabel Cristina Garcia Moreno, Juan David Serna Lorza, Daniela Hincapie-Ayala, Maria Camila Diaz, Edgar Darío Alzate Gallego, Juan Pablo Sanchez Sanchez

**Affiliations:** ^1^ Departamento de Cirugía Pediátrica, Fundación Valle del Lili, Cali, Colombia; ^2^ Centro de Investigaciones Clínicas, Fundación Valle del Lili, Cali, Colombia; ^3^ Facultad de Ciencias de la Salud, Universidad Icesi, Cali, Colombia; ^4^ Departamento de Patología, Fundación Valle del Lili, Cali, Colombia

**Keywords:** appendicitis, intestinal diseases, parasitic, pediatrics, Balantidiasis, case report

## Abstract

**Introduction:**

Acute appendicitis secondary to parasitic infections is uncommon, being detected in less than 1% of cases. Balantidium coli is a parasite found in pigs and primates with zoonotic potential. To date, only three cases of acute appendicitis induced by this parasite have been documented globally.

**Case:**

A 7-year-old female patient, who consumed pork daily, presented to the emergency department with a one-day history of abdominal pain in the lower quadrants, described as colic-like, alongside abdominal distension. Initial abdominal radiography led to a diagnosis of intestinal obstruction. Conservative management without therapeutic response necessitated referral to a higher complexity center. Upon admission, an abdominal computed tomography scan diagnosed acute appendicitis and secondary ileus. During surgical intervention, an appendiceal phlegmon formed by loops of the small intestine was mechanically released, revealing a perforated appendix with extensive fecal peritoneal contamination. Pathological analysis identified an inflammatory infiltrate and the presence of Balantidium coli trophozoites. Medical treatment included Piperacillin-Tazobactam and Metronidazole. The patient was discharged after 10 days of medical treatment.

**Discussion:**

Acute appendicitis caused by Balantidium coli is a rare occurrence. It is crucial to identify parasites in pathological samples due to their impact on postoperative management. The close contact between humans and pigs, especially in developing countries, suggests that the prevalence of parasitic infection and colonization by Balantidium coli may be higher than currently recognized. Regarding the identification of this patient's specific exposure, the regular consumption of pork suggests the hypothesis that improper processing is linked to the acquisition of the parasitic infection.

## 1 Introduction

Acute appendicitis represents one of the most common surgical emergencies worldwide, predominantly affecting individuals in their second and third decades of life (1). The lifetime incidence is estimated to be 7%–8%, with an annual rate of 90–100 cases per 100,000 individuals in developed countries (1). Despite its widespread occurrence, the precise causes and risk factors for acute appendicitis remain the subject of ongoing research. Parasitic infections, though rare, are identified as a causative agent in close to 1% of cases (2). Prominent among the associated parasites are Ascaris lumbricoides, Trichuris trichiura, Enterobius vermicularis, Entamoeba histolytica, Giardia duodenalis, and various tapeworms (3).

Balantidium coli, a parasite primarily affecting pigs, primates, and humans, exhibits significant zoonotic potential that facilitates its transmission through the consumption of water and food contaminated with its cysts (4). A significant correlation has been established between infection by this protozoan and proximity to pigs and their waste (4). In humans, Balantidium coli typically colonizes the cecum and colon, although cases of extraintestinal infections have also been observed. Clinical manifestations in humans vary, ranging from intermittent diarrhea to abdominal pain and dysentery (4). Only three cases of acute appendicitis caused by this parasite have been reported, characterized by their severity and lack of survival (5–7). We present the fourth case, notable for being the first with a successful recovery following perforation and peritoneal contamination, induced by a Balantidium coli infection in an academic hospital setting.

## 2 Case

A 7-year-old female patient, residing in an urban area and with no prior medical history, was brought to the emergency department by her parents. The patient had a history of consuming pork daily. Clinical symptoms had begun the day before admission, characterized by abdominal pain in the lower quadrants of a colic-like nature, accompanied by abdominal distension. Additionally, the patient exhibited fever, multiple vomiting episodes, and diarrhea. She had been self-medicated with an antidiarrheal (Loperamide) in an unspecified dose by her parents.

Laboratory tests revealed leukocytosis (13,160 × 10^3^/μl) without neutrophilia and thrombocytosis (554,000 × μl). Urinalysis showed no pathological findings. Imaging evaluation included an abdominal radiography, which reported the presence of multiple air-fluid levels and absence of gas in the distal segment, suggesting an intestinal obstruction. Given these findings, management with Ampicillin/Sulbactam was initiated, and she was transferred to a higher complexity center on the third day of the onset of symptoms.

**Figure 1:**
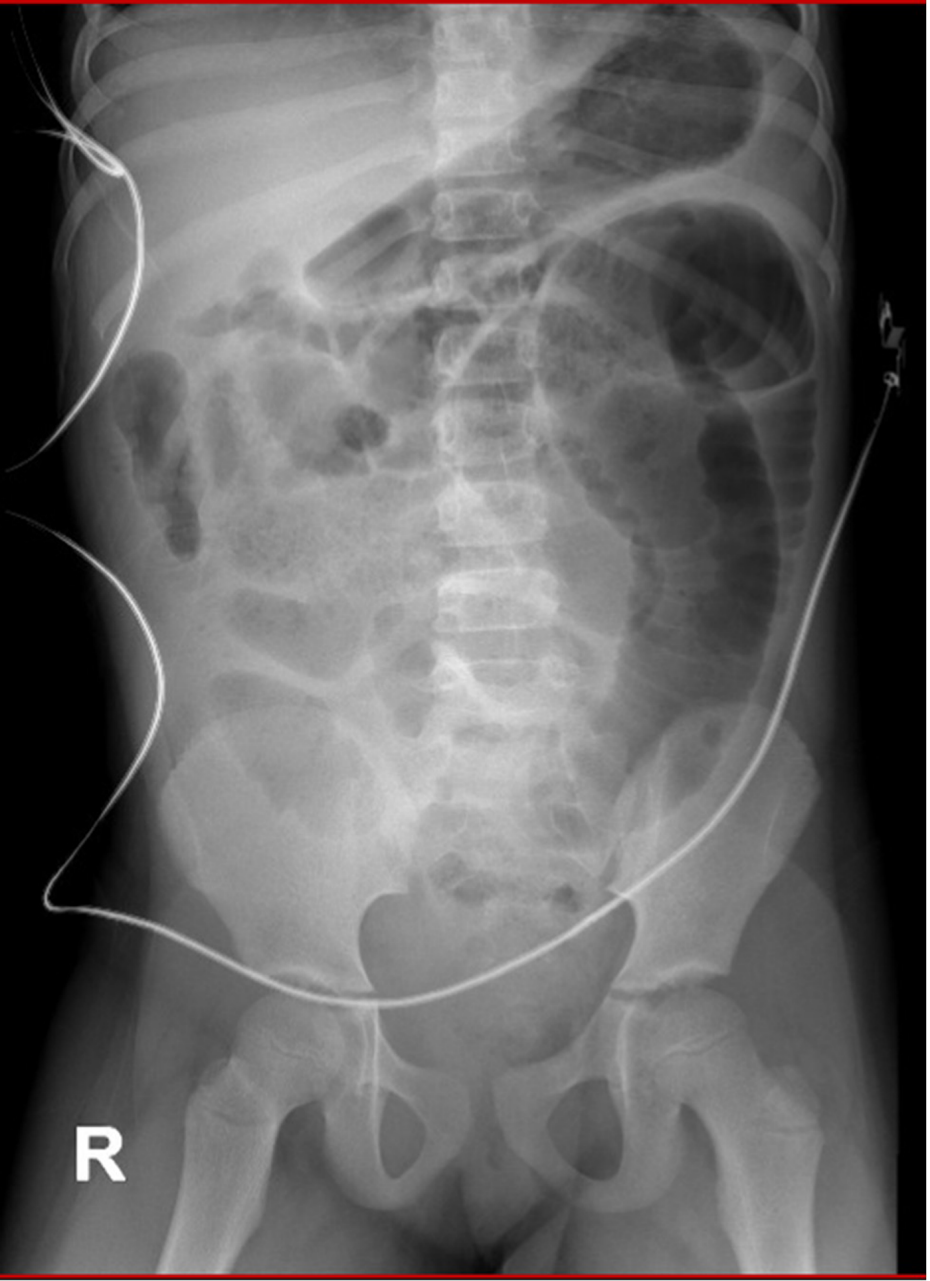
Abdominal radiography after admission to the higher complexity institution showing coprostasis and distension of the small bowel without evidence of air-fluid levels.

Upon admission to the institution, the patient exhibited signs of dehydration, tachypnea, a restrictive respiratory pattern, a distended and tympanic abdomen with pain upon palpation in the lower abdomen. Additionally, she presented with fever, multiple vomiting episodes of food content, and diarrheal stools without the presence of mucus or blood. Resuscitation management was initiated, and she was assessed by the pediatric surgery department, which found no signs of peritoneal irritation on physical examination. A rectal examination revealed the presence of abundant fecal matter in the rectal ampulla, leading to the placement of a nasogastric tube and administration of an enema with 175 ml of isotonic saline solution and 75 ml of soapy solution, resulting in minimal fecal return. Post-enema abdominal radiography showed coprostasis and distension of the small bowel without evidence of air-fluid levels (Figure 1). Admission laboratory tests showed leukocytosis (16,630 × 10^3^/μl) with an increase in neutrophils (12,940 × 10^3^/μl), thrombocytosis (478,000 × μl), and elevated levels of C-reactive protein (35.06 mg/dl, with a reference range of 0–5 mg/dl). Arterial gases revealed compensated respiratory alkalosis and hypokalemia (3.2 mEq/l). Given the inadequate response to medical treatment for intestinal obstruction, treatment was escalated to Piperacillin/Tazobactam, and the patient was transferred to the intensive care unit.

**Figure 2:**
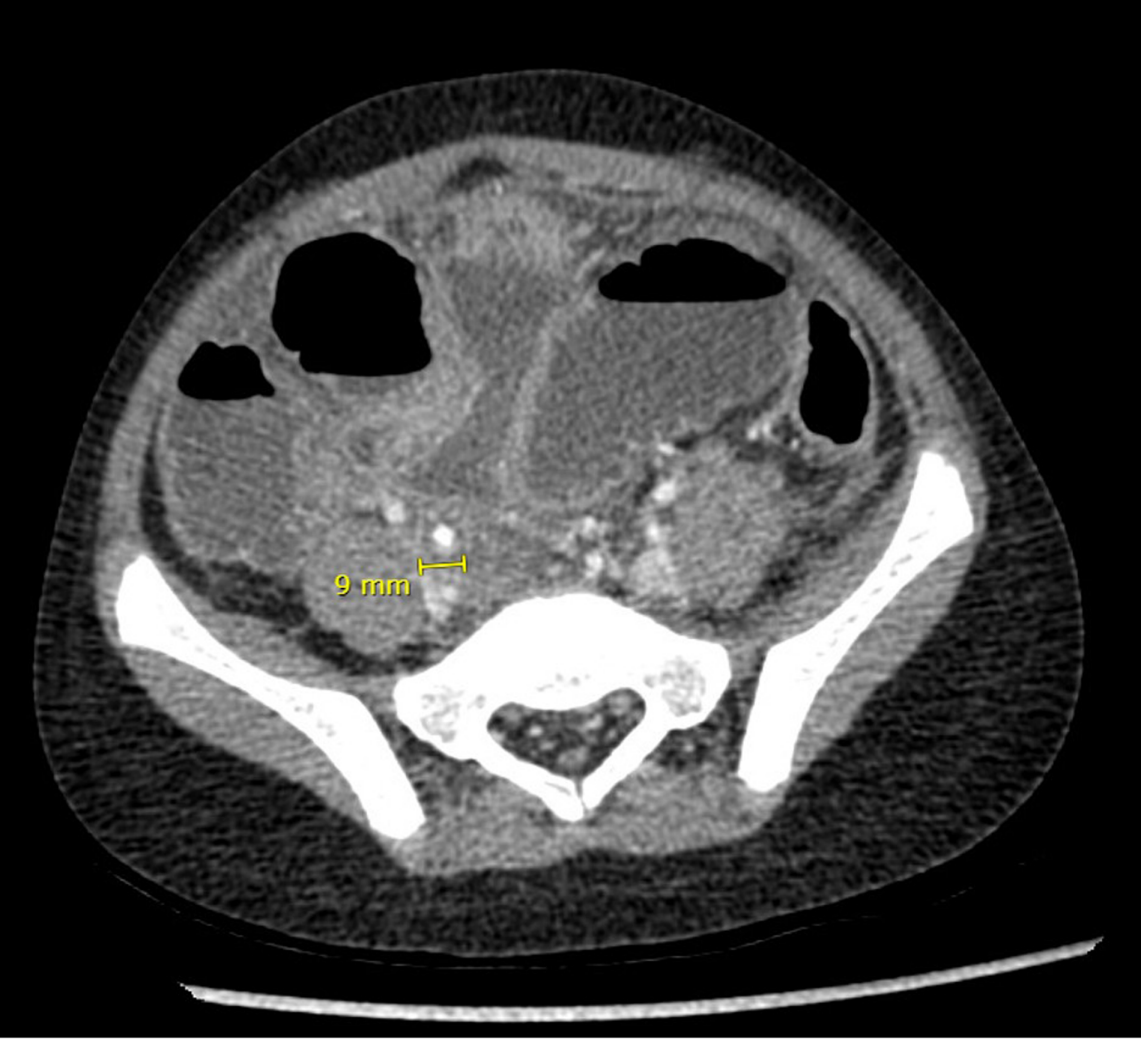
Abdominal CT scan performed on the patient after failure of clinical improvement with medical management.

Given the lack of response to medical treatment, a decision was made to perform an abdominal computed tomography (CT) scan on the first day of admission to the institution. The images revealed notable dilatation of the small bowel, with a maximum diameter of 4.3 cm, without evident transition zones, and an appendix measuring 9 mm with increased enhancement of its walls, presence of gas within, surrounding peritoneal thickening, free abdominal fluid, and pneumoperitoneum (Figure 2). These findings led to the diagnosis of acute appendicitis with secondary ileus. The patient was prepared for surgical intervention. The procedure, initially planned as a laparoscopic appendectomy, had to be converted to an open surgery via a midline infraumbilical laparotomy due to the development of respiratory distress caused by the insufflation of pneumoperitoneum and limited visibility due to intestinal distension. During the surgical procedure, an appendiceal phlegmon formed by loops of the small intestine was mechanically released without requiring intestinal resection. The appendix was found in a subcecal position, necrotic, and perforated at its tip. Given the extensive fecal contamination, a peritoneal irrigation was performed until clear fluid return was achieved.

In the postoperative period, the patient was taken to the ICU, where she continued antibiotic management with Piperacillin/Tazobactam. The use of vasopressors or ventilatory support was not necessary. She remained with ileus for six days, necessitating the use of parenteral nutritional support. Control laboratory tests performed on the fourth postoperative day showed improvement in inflammatory markers, with a reduction in the levels of leukocytes (12,980 × 10^3^/μl), neutrophils (7,300 × 10^3^/μl), and C-reactive protein (7.94 mg/dl). The patient was then transferred to general ward care. Resolution of the ileus and tolerance to oral intake were achieved by the sixth postoperative day.

**Figure 3:**
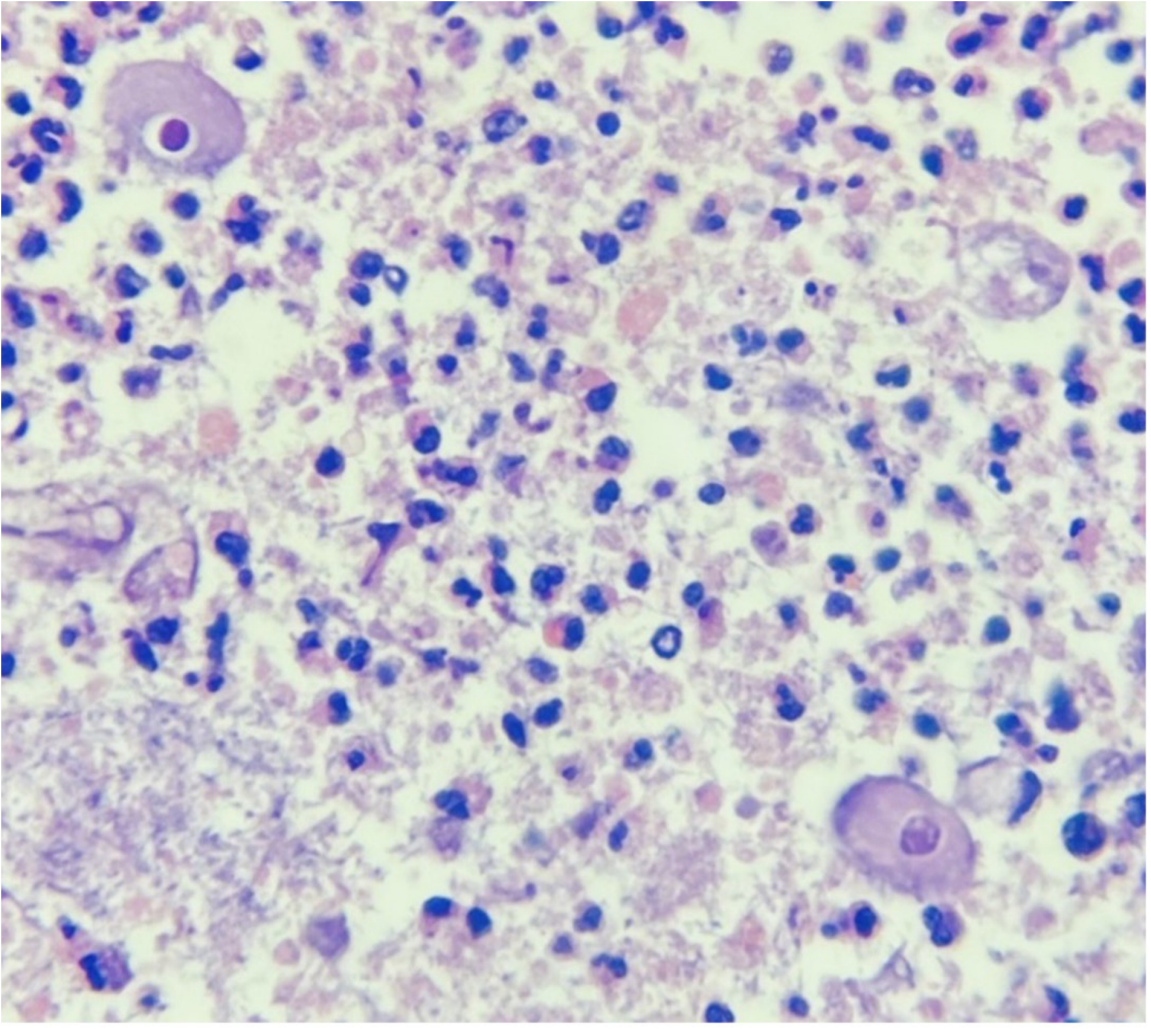
Pathologic analysis of the surgical specimen showing Balantidium coli trophozoites. Hematoxylin and Eosin staining, original magnification ×400.

**Figure 4:**
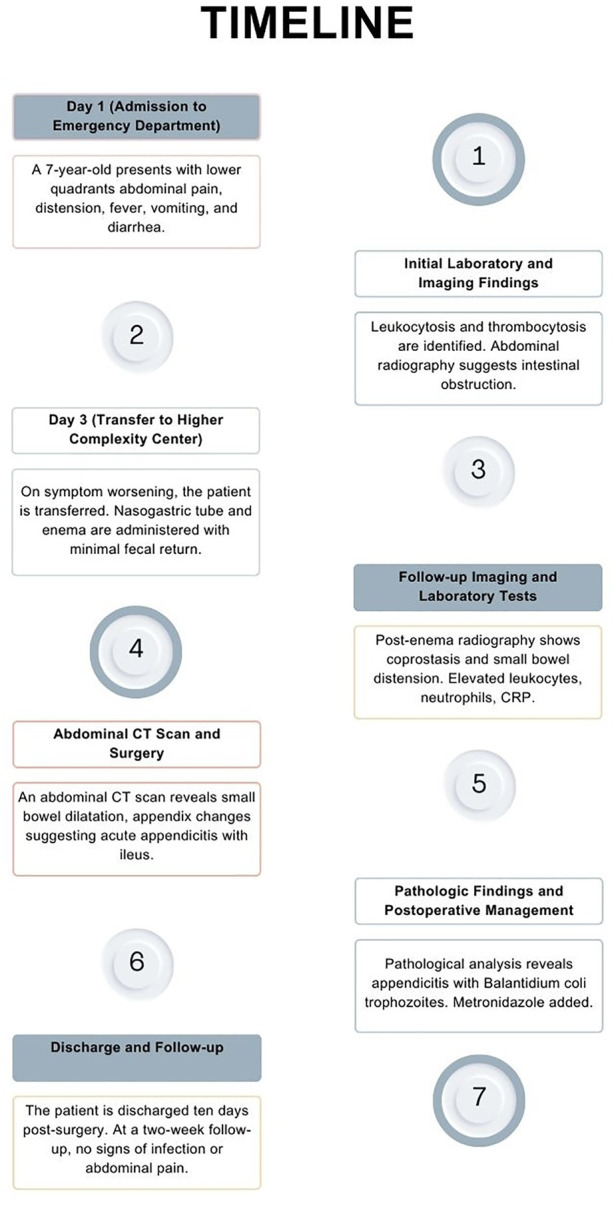
Timeline depicting all relevant events in the patient's clinical course.

Pathologic analysis revealed an inflammatory infiltrate and perforation of the appendix with liquefactive necrosis, along with the presence of Balantidium coli trophozoites, leading to the definitive diagnosis of acute appendicitis caused by Balantidium coli (Figure 3). Following this finding, Metronidazole was added to the medical management, administered at a dose of 500 mg every 8 h. The postoperative recovery proceeded without complications. She completed treatment with 10 total days of Piperacillin/Tazobactam and 7 days of Metronidazole. Ten days after the surgical intervention, she was discharged. At the two-week outpatient follow-up no signs of infection in the surgical wound were seen, adequate wound healing was observed, and no abdominal pain was present (Figure 4).

## 3 Discussion

Cases of Balantidium coli infections are anecdotal, and extensive studies focused on the etiology of appendicitis in numerous patients, including those investigating parasites as a possible cause, have not documented cases of this nature (8, 9). Therefore, the report of the fourth confirmed case of acute appendicitis caused by Balantidium coli, which is also the first to present complications such as perforation, extensive abdominal contamination, and generalized peritonitis with survival, represents a contribution to the existing body of evidence (5–7).

Parasitic infections are a recognized, though infrequent, cause of appendicitis, accounting for less than 1% of all cases in previous studies in other developing countries (2). The pathophysiology behind appendicitis suggests that obstruction of the appendiceal lumen by protozoans or cysts is rare, due to the size of the parasitic organisms, which generally range between 50 and 200 μm in length (10). However, partial obstructions together with local inflammatory processes caused by the infection are considered to lead to lumen obstruction (8). Among the most common parasites causing infections in humans and associated with appendicitis are Enterobius vermicularis, Ascaris lumbricoides, Giardia lamblia, and Strongyloides stercoralis, which are responsible for 25%, 8%, 3%, and 1% of parasite-related appendicitis cases, respectively (9).

Balantidium coli is distinguished as the only ciliated parasite capable of infecting humans, which can be histologically identified in two forms: cyst and trophozoite (11). This unique morphological characteristic facilitates its differentiation from other pathogenic microorganisms (11). In its cyst phase, it measures between 40 and 60 μm, while in the trophozoite phase, it can reach a diameter of 60–100 μm (12). This size difference is significant when compared to the trophozoites of Entamoeba histolytica, which have a morphological similarity but whose dimensions range between 10 and 20 μm (12). Regarding the identification of this patient's specific exposure, although exposure to water or vegetables contaminated by cysts is not evident, given her urban setting with no direct proximity to pigs, the regular consumption of pork suggests the hypothesis that improper handling or processing of this meat could be linked to the acquisition of the parasitic infection.

Early identification of Balantidium coli and other parasites in pathological samples is crucial, as it raises important questions that must be addressed to ensure proper postoperative management. The approach to antiparasitic treatment in cases of appendicitis where the etiology is not parasitic, but parasites are incidentally found, remains an area of discussion. Such decisions are usually based on the presence or absence of symptoms of parasitic infection. Although the lack of comparative studies limits definitive conclusions, it is recommended, based on clinical experience, to initiate antiparasitic treatment upon the histopathological finding of parasites (8). This follows the logic that while appendectomy resolves an immediate complication, it does not address the underlying causative agent. In this case, the decision to initiate treatment with Metronidazole was guided by its effectiveness against the causative agent, the symptomatology of the parasitic infection, and the significant abdominal contamination. Although Balantidiasis can also be treated with tetracyclines or iodoquinolol, Metronidazole was chosen due to its alignment with current practices for managing extensive peritoneal fecal contamination while simultaneously treating Balantidium coli (13, 14).

The practice of performing peritoneal irrigations in adult patients has been associated with a reduction in the formation of intra-abdominal abscesses following cases of appendicular perforation (15). The rationale for the use of peritoneal irrigations is based on the hypothesis that decreasing the bacterial load should reduce the incidence of intra-abdominal abscesses, facilitate the early resolution of peritonitis, and consequently improve the postoperative prognosis (15). However, the application of this procedure in pediatric patients, who exhibit high rates of intra-abdominal abscesses, remains a topic of debate. While some studies have shown a decrease in the rates of postoperative intra-abdominal abscesses, from 17.2% to 4.0%, others have not demonstrated benefits in the duration of hospital stay or in the development of complications, at the expense of an increase in surgical time compared to suction alone (15, 16). Nonetheless, it is crucial to distinguish between patients with localized peritonitis and those with diffuse purulent peritonitis. Under this context, an extensive washout until achieving a completely clean abdomen may still be a viable option, as was done in this patient (17).

In conclusion, acute appendicitis caused by Balantidium coli is an extremely rare event. The absence of complications typically associated with appendicular perforation, such as intra-abdominal abscesses, cannot be directly linked to the specific treatment strategy chosen here, mainly due to the lack of comparative data resulting from the rarity of this causative agent. Despite this, the therapeutic approach adopted, facilitated a satisfactory outcome in a complex case that in similar cases has previously led to lethal outcomes. Furthermore, considering that pigs and primates are usually carriers of this parasite, the proximity between humans and pigs, especially in developing countries, suggests that the prevalence of infections and colonization by Balantidium coli might be underestimated.

## Data Availability

The original contributions presented in the study are included in the article/Supplementary Material, further inquiries can be directed to the corresponding author.
